# A dosimetric model for the heterogeneous delivery of radioactive nanoparticles *In vivo*: a feasibility study

**DOI:** 10.1186/s13014-017-0794-z

**Published:** 2017-03-17

**Authors:** Andrew B. Satterlee, Peter Attayek, Bentley Midkiff, Leaf Huang

**Affiliations:** 1 Division of Molecular Pharmaceutics and Center for Nanotechnology in Drug Delivery, Eshelman School of Pharmacy, University of North Carolina at Chapel Hill, Chapel Hill, NC 27599-7571 USA; 2 UNC and NCSU Joint Department of Biomedical Engineering, Chapel Hill, NC 27599 USA; 3 Translational Pathology Laboratory, University of North Carolina at Chapel Hill, Chapel Hill, NC 27599-7571 USA

**Keywords:** Dosimetry, Heterogeneous, Tumor, Nanoparticle, Internal Radiation Therapy

## Abstract

**ᅟ:**

Accurate and quantitative dosimetry for internal radiation therapy can be especially challenging, given the heterogeneity of patient anatomy, tumor anatomy, and source deposition. Internal radiotherapy sources such as nanoparticles and monoclonal antibodies require high resolution imaging to accurately model the heterogeneous distribution of these sources in the tumor. The resolution of nuclear imaging modalities is not high enough to measure the heterogeneity of intratumoral nanoparticle deposition or intratumoral regions, and mathematical models do not represent the actual heterogeneous dose or dose response. To help answer questions at the interface of tumor dosimetry and tumor biology, we have modeled the actual 3-dimensional dose distribution of heterogeneously delivered radioactive nanoparticles in a tumor after systemic injection.

**Methods:**

24 h after systemic injection of dually fluorescent and radioactive nanoparticles into a tumor-bearing mouse, the tumor was cut into 342 adjacent sections and imaged to quantify the source distribution in each section. The images were stacked to generate a 3D model of source distribution, and a novel MATLAB code was employed to calculate the dose to cells on a middle section in the tumor using a low step size dose kernel.

**Results:**

The average dose calculated by this novel 3D model compared closely with standard ways of calculating average dose, and showed a positive correlation with experimentally determined cytotoxicity *in vivo*. The high resolution images allowed us to determine that the dose required to initiate radiation-induced H2AX phosphorylation was approximately one Gray. The nanoparticle distribution was further used to model the dose distribution of two other radionuclides.

**Conclusions:**

The ability of this model to quantify the absorbed dose and dose response in different intratumoral regions allows one to investigate how source deposition in different tumor areas can affect dose and cytotoxicity, as well as how characteristics of the tumor microenvironment, such as hypoxia or high stromal areas, may affect the potency of a given dose.

**Electronic supplementary material:**

The online version of this article (doi:10.1186/s13014-017-0794-z) contains supplementary material, which is available to authorized users.

## Background

Quantitative radiation dosimetry requires knowledge of both patient anatomy and distribution of the absorbed dose. Improvements in dosimetric models have been seen not only in external beam radiation therapy, but also in dose planning for several types of internal radiation therapy, including in brachytherapy [1, 2], selective internal radiation therapy [3, 4], and radioimmunotherapy [5–7]. Accurate dosimetry for internal radiation therapy can be especially challenging, given the heterogeneity of patient anatomy, tumor anatomy, and source deposition. As radiotherapy sources have become smaller and their deposition less controlled—from brachytherapy seeds to ^90^Yttrium (^90^Y) microspheres to radionuclide-chelated monoclonal antibodies administered systemically—more accuracy is needed to determine the increasingly heterogeneous distribution of the sources, and how this distribution affects therapeutic outcomes. Because of this, a huge amount of effort has been invested in the creation of dosimetric algorithms to analyze dose deposition and dose–response in clinical and preclinical models. Some of these models are based on theoretical calculations that make educated assumptions about source deposition [8–11], while others use nuclear imaging methods such as single photon emission computed tomography (SPECT) to approximate the source distribution [4, 5, 12]. Although theoretical models can help one quickly change parameters and estimate outcomes, the calculated distributions do not represent the actual heterogeneity of source and dose distribution in a tumor. While some preclinical SPECT scanners have been accepted as quantitative [13] and achieved sub-millimeter spatial resolution [14, 15], the gamma camera on clinical scanners is only ~10 mm [16]. Positron emission tomography (PET) is known to have greater sensitivity than SPECT, but its resolution is bound to the energy of the positron itself as well as to the detector limits [17]. Positrons with higher energy at the time of ejection travel a longer distance from the source before their detectible annihilation reaction, resulting in an additional contribution to spatial resolution of over 0.5 mm for [18] Fluorine and greater than 6 mm for ^82^Rubidium [17]. In either modality, the reconstructed images are not near cellular resolution, and may have trouble distinguishing source deposition within different tumor areas.

We have generated a high resolution dose map with a voxel size of 10 μm^3^ from an actual 3-dimensional (3D) intratumoral distribution of systemically delivered radioactive nanoparticles, and have used this dose map to quantify the dose in Gray (Gy) to each cell in a section of the tumor. We have also successfully correlated increasing dose with increasing DNA double-stranded break repair via phosphorylation of H2AX, an enzyme that is phosphorylated in response to DNA double-stranded breaks, and is the most representative marker of DNA damage caused by ionizing radiation [18]. The following represents a proof-of-concept that this type of high-resolution correlation is possible and may provide ways to strengthen our understanding at the interface of biology and dosimetry.

## Methods

### Materials

^177^Lutetium Chloride (^177^LuCl_3_) was purchased from PerkinElmer (Waltham, MA). N-(Carbonyl-methoxypolyethyleneglycol 2000)-1,2-distearoyl-sn-glycero-3-phosphoethanolamine, sodium salt (DSPE-PEG2000) was purchased from NOF America Corporation (White Plains, NY). DSPE-PEG2000-Anisamide (DSPE-PEG-AA) was synthesized in our lab as described previously [19]. 1,2-dioleoyl-*sn*-glycero-3-phosphate (DOPA) and 1,2-dioleoyl-3-trimethylammonium-propane (DOTAP) were purchased from Avanti Polar Lipids (Alabaster, AL). Primary antibody against p-H2AX was purchased from Cell Signaling (Rabbit mAb #9718). Anti-rabbit secondary antibody was purchased from Cellsignaling (#4414). Other chemicals were purchased from Sigma-Aldrich (St. Louis, MO) or ThermoFisher (Waltham, MA).

### Cell lines

UMUC3 human bladder cancer cells and NIH/3T3 (3T3) murine fibroblasts were used in the described experiments. UMUC3 cells were grown in Dubelcco’s Modified Eagle’s Medium (DMEM) supplemented with 10% fetal bovine serum and 1% penicillin/streptomycin. 3T3 cells were grown in DMEM supplemented with 10% bovine calf serum and 1% penicillin/streptomycin.

### Experimental animals

Female athymic nude mice aged six to eight weeks were used for all experiments. The mice were purchased from the National Cancer Institute (Bethesda, MD) and bred at the Division of Laboratory Animal Medicine at the University of North Carolina-Chapel Hill. All work performed on these animals was approved by the Institutional Animal Care and Use Committee at the University of North Carolina-Chapel Hill, protocol #14-045.

### DiI-^177^Lu-LCP fabrication

Fabrication of the ^177^Lu-loaded Lipid-Calcium-Phosphate (LCP) nanoparticle (^177^Lu-LCP) is well characterized and has been described previously [20–23]. In brief, ^177^Lu was co-precipitated with calcium, phosphate, and the phospholipid DOPA in a reverse microemulsion. The resulting nanoparticle core was dissolved in chloroform and the outer leaflet lipids DOTAP, cholesterol, and DSPE-PEG-AA were added to the chloroform solution. The lipophilic dye DiI was also added to the chloroform before evaporating the solution into a lipid film on the slides of a glass vial. Adding water to hydrate the lipid film, followed by vortexing and sonication, allowed the free lipids to arrange themselves around the nanoparticle cores and form a lipid bilayer that was soluble in water. The DiI accumulated in the lipophilic bilayer of the LCP nanoparticles, and excess DiI was removed using sucrose gradient centrifugation. DiI was chosen as a marker for LCP because it has previously been shown to be a faithful label of lipid bilayers [24].

### ^177^Lu-LCP pharmacokinetic study

^177^Lu-LCP was injected intravenously into n = 5 female athymic nude mice in a 10% w/v sucrose/water solution. At 0.5, 1, 2, 4, 8, and 24 h after injection, ~20 μL of blood was collected from each mouse and measured for ^177^Lu using gamma scintillation. This value was used to estimate the amount of ^177^Lu remaining in circulation, and therefore the amount of ^177^Lu in the tumor, assuming that the tumor accumulation was proportional to the fraction of the injected dose that had left circulation at that time, with the maximum tumor accumulation occurring at t = 24h and a total blood volume per mouse of 1.8 mL. ^177^Lu-LCP was formulated with DOTAP in the outer leaflet in order to decrease circulation time and expedite tumor accumulation of the nanoparticles. Time dependent tumor accumulation was used for all dosimetry models herein. The rationale for this method is expanded in Additional file 1, and data is presented in Additional file 2: Table S1 and Additional file 3: Figure S1.

### Standard dose calculations (MIRD and single point source)

We used two separate methods to help justify our multi-point source dosimetric approach. The Medical Internal Radiation Dose (MIRD) formula was used to calculate the dose to the tumor assuming homogeneous source delivery. The full derivation of the MIRD dose is included in Additional file 1. In summary, the standard equation $\overline{D}={\displaystyle {\int}_0^{\infty}\overset{\bullet }{D}}(t) d t$ was used to calculate the dose of ^177^Lu to the tumor [3, 6, 7, 25, 26].

Dose kernels for point sources in an infinite water medium [27–29] were also used to approximate the dose, assuming that all activity was contained in a single point source and that the absorbed fraction was one. The dose at r = 0 was adjusted as described in the next section to avoid an asymptotic rise in dose at the source. The dose calculated from MIRD and by kernel values was comparable (Additional file 4: Table S2) and supports the use of dose kernels for this new multi-point source model.

Decay of ^177^Lu and the kinetics of ^177^Lu-LCP accumulation in the tumor were accounted for within these calculations.

### Novel dose kernel calculations (multi-point source)

In short, these novel dose calculations aim to determine the dose to each cell in a tumor section by calculating the activity present in each 10 μm^3^ voxel within a 3D reconstructed tumor. Each voxel is treated as its own point source with its own set of dose kernels. For our dose kernel calculations, the density of tumor tissue was assumed to be that of water, and would therefore not perturb the kernel values or dose deposition. Concentration of LCP nanoparticles in the tumor was calculated to be approximately ten parts per million (weight:weight); it was assumed that these nanoparticles also did not perturb the kernel calculations. Dose kernels were interpolated so that the length of each kernel was 10 μm (Additional file 4: Table S3 and Additional file 5: Table S4). This did not change the total absorbed dose with respect to the un-interpolated kernels. It is known that dose kernels can lose fidelity at small distances due to the inverse square law (explained in the publication also providing the kernels themselves [27]), so to avoid an unrealistic asymptotic rise in dose deposition at distances close to r = 0, values at small r were set so that those rates would decrease exponentially with increasing r. In this way, the total absorbed dose of both the interpolated and un-interpolated kernels were comparable to the dose calculated by the MIRD formula, as shown in Additional file 4: Table S2.

To estimate the dose absorbed by each cell nucleus from a given point source, the average number of nuclei in each annular kernel volume (annular volume = 4/3πr_2_^3^-4/3πr_1_^3^, with r_2_-r_1_ = 10 μm) was calculated using an actual fluorescent image of the nuclear distribution in the tumor (Additional file 6: Figure S3). From this image, the volume fraction occupied by all cell nuclei was 0.4, and the average size of a cell nucleus was ~ 10 μm^3^. To calculate the statistical dose to each nucleus in each annulus from a point source, the total dose deposited in an annulus was divided by the number of nuclei in that annulus and multiplied by the average volume fraction occupied by nuclei in the tissue. The dose per nucleus was calculated because our fluorescent images did not clearly define cell boundaries and what fraction of the tumor consisted of extracellular matrix, etc.

### Microdosimetry

Intratumoral nanoparticle distribution was quantified by first formulating DiI-^177^Lu-LCP (described above) and systemically injecting these radioactive and fluorescent particles into the tail vein of a UMUC3/3T3 tumor-bearing mouse. At t = 24 h after treatment, the tumor was dissected, fixed, and frozen in OCT. The frozen tumor was then sectioned into 342 adjacent sections, each 10 μm thick, which were mounted on slides and stained with DAPI. Each section was individually imaged for DAPI and DiI fluorescence at the Translational Pathology Lab at The University of North Carolina at Chapel Hill (UNC) with the Aperio Versa 200 digital pathology scanner (Leica Biosystems Richmond, Inc., USA), which digitally scanned the entire tumor section on each slide with a resolution of 0.32 μm/pixel. Each section was examined for artifacts such as folding or breakage of the section; slides with major artifacts were replaced with a duplicate of their adjacent section (only 24 out of 342 slides were replaced). Slight creasing in some sections also caused small areas of falsely positive DiI signal, which were removed by setting the value of the bright pixels along the crease to zero. For each 8-bit grayscale image, background subtraction was set to remove all noise at intensity ≤ 20 (out of 255).

Five slides—#003, 301, 302, 303, and 340—were imaged before all others and were used to generate a correlation between DiI signal and radioactivity in the tissue. Imaging all 342 slides took a significant amount of time, but by quickly imaging these select slides while the radioactivity was still high enough to be measured using liquid scintillation counting, we could determine this correlation. After these slides had been imaged for their DiI distribution, their coverslips were removed and the tissues were wiped off using a small piece of Kimwipe. Any residual tissue was collected by using two additional Kimwipes to wipe the slide. Each tissue, now on a Kimwipe, was placed in a 7 mL glass scintillation vial filled with 4 mL of Ultima Gold scintillation cocktail. After vortexing and sonicating each vial to saturate the tissue with scintillation fluid, the vials were read for radioactivity using a liquid scintillation counter and compared against a calibration curve of known amounts of ^177^Lu radioactivity. The amount of radiation per tissue sample, in Bq, was adjusted for six days of decay between injection of nanoparticles and this quantification. The total amount of DiI fluorescence in each of these samples was also quantified as the total number of relative fluorescence units (RFU) per section. The Bq per RFU in each of the five sections was calculated and averaged to be 5.3E-5 ± 1.5E-5 (Avg ± SD). This factor was then used to calculate the total radioactivity in all 342 samples.

The DiI distribution files for each of the 342 sections were converted to TIFF images and downsampled so that each pixel was ~10 μm^2^. Now each pixel represented a 10 μm^3^ voxel due to the 10 μm thickness of each section. All sections were then registered using the StackReg plugin in the FIJI image processing software so that all images could be converted to a 3D stack that represented the 3D tumor. StackReg rotated and translated each section to co-localize its outline and features with the adjacent section in the stack. A middle section in the stack, section 171, was the section on which the dose map was calculated.

A novel algorithm was created in MATLAB and executed on the University of North Carolina’s Killdevil computing cluster to create the dose distribution map describing the cumulative deposited dose from all voxels in all sections onto section 171. The algorithm measured the (x,y,z) coordinates for all 10 μm^3^ voxels in the stack and individually determined the straight line distance (r) from each voxel to every voxel in section 171. The dose contribution corresponding to each distance (r) was calculated using our interpolated dose kernel values and adjusted to reflect the concentration of DiI-^177^Lu-LCP in the voxel (as described in the previous paragraph). In this way, each voxel in the stack was treated as a distinct source with an activity calculated from its fluorescence intensity and a dose distribution based on the dose kernel.

### p-H2AX quantification

To further validate our dosimetry method, and also to quantify the dose required to initiate an increased rate of DNA damage repair, section 171 was stained for the DNA double-stranded break repair enzyme p-H2AX using immunofluorescence. After imaging the DiI distribution in section 171, the coverslip was removed and the section was re-stained and re-imaged. The dose map was then overlaid on the p-H2AX distribution map in order to correlate absorbed dose with p-H2AX expression (Fig. 4).

### Quantifying dose distributions for other nuclides

Dose kernels [27] for ^33^Phosphorus (^33^P) and ^90^Y were used to calculate the dose distribution of these nuclides from the same 3D nanoparticle (source) distribution used for ^177^Lu. The dose kernels for ^33^P and ^90^Y were interpolated and used to create a new overall dose map onto section 171. In this way, we could compare differences in dose distributions from a single source distribution. Results are provided in Fig. 5.

### Calculating radial dose distribution

In order to measure what fraction of the total dose to a cell was contributed by radiation sources at different distances away from that cell, a completely separate and hypothetical source distribution was used. We generated a 3D field of homogeneous radioactivity and used the interpolated dose kernels for ^177^Lu, ^90^Y, and ^33^P to calculate the dose contribution onto one 10 μm^3^ voxel (a “cell”) from each 10 μm-thick spherical annulus emanating outward. Results are provided in Fig. 6.

## Results

To generate the tumor for our model, a nude athymic mouse bearing a UMUC3/3T3 tumor [20, 30] was treated with lipid-calcium-phosphate nanoparticles that were loaded with ^177^Lu and labeled with fluorescent DiI (DiI-^177^Lu-LCP). Twenty-four h after treatment, the tumor was dissected and weighed. At this time, the 0.13 g tumor was measured to contain 60.3 kBq of ^177^Lu using gamma scintillation. The dose to the tumor at each time point between t = 0 and t = 24 h was adjusted to reflect radioactive decay, as well as tumor accumulation over time as described in the Additional file 2: Table S1 and Additional file 3: Figure S1 pharmacokinetic studies.

**Fig. 1:**
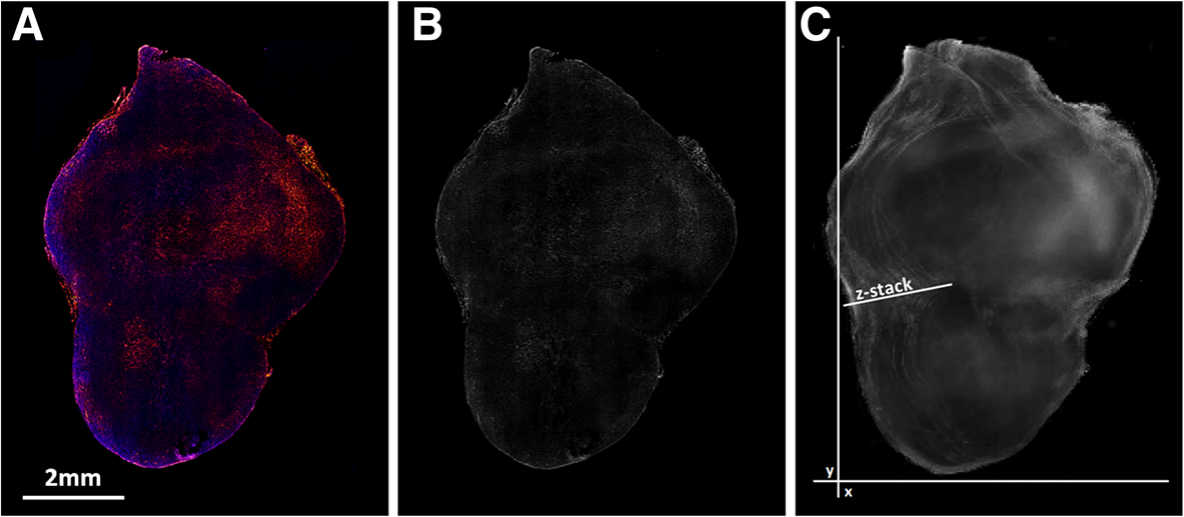
Intratumoral DiI-^177^Lu-LCP Distribution: **a**) two-channel image of DAPI nuclear stain (*blue*) and DiI (*red*) in section 171; **b**) Isolated and background-subtracted DiI signal; **c**) 3D stack of 342 adjacent tumor sections



The tumor in question was fixed, frozen, sectioned into 342 adjacent sections, and imaged to quantify DiI-^177^Lu-LCP distribution (red color, Fig. 1a). The DiI channels for each image (Fig. 1b) were registered and stacked to create a 3D model (Fig. 1c) of the tumor’s DiI distribution. A rendering of this 3D model is provided as a video in Additional file 7: Figure S4.

**Additional file 7:** Figure S4: 3D Rendering of Stacked Sections to Create 3D Model. (MP4 805 kb)


In order to determine if DiI distribution could indeed be used to model ^177^Lu distribution, five of the imaged tumor sections were wiped from their slides and read using liquid scintillation counting against a calibration curve of free ^177^Lu. The total radioactivity in each of these imaged sections was divided by each section’s total fluorescence to arrive at an average value for Bq/RFU of 5.27E-05 (using time t = 0 to account for decay). Applying this factor to all sections yielded a total of 52.9 kBq in all sections combined at t = 24 h, which was ~88% of the total activity in the intact tumor. The slight discrepancy in total radiation is due at least in part to the front and back ends of the tumor not being included in the 342 sections used to create this model. Therefore, it was reasonable to use the DiI distribution as a surrogate marker for the 3D distribution of ^177^Lu in the tumor.

**Fig. 2:**
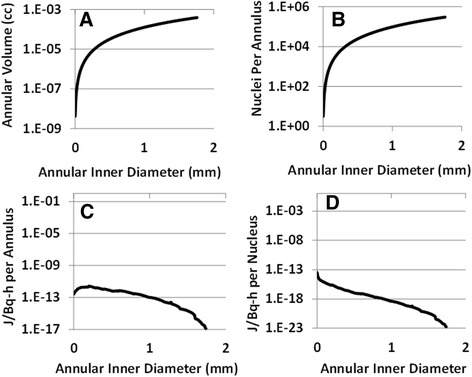
Dose Kernel Distribution; data recorded every 0.01 mm: **a**) Volume of each annulus with inner diameter on x-axis; **b**) Number of nuclei per annulus as calculated in Methods section; **c**) Total absorbed dose per annulus in J/Bq-h; **d**) Absorbed dose per nucleus in J/Bq-h

At this point, the ^177^Lu concentration in every 10 μm^3^ voxel in the 3D DiI model was known, and each voxel was considered its own point source. Using the interpolated dose kernels shown in Additional file 4: Table S3, a dose map was created onto a middle section in the stack: section 171. A novel MATLAB code (described in Methods) was used to calculate the distance—and therefore the dose contribution—of each voxel in each section to each voxel in section 171. Figure 2 shows how four different parameters change as distance from the source, and therefore the annular inner diameter, increases. Annular volume and nuclei per annulus (A and B) both increase exponentially. Absorbed dose per annulus increases slightly at short distances before dropping, while dose per nucleus shows a sharp decrease at short distances followed by a continued decrease (C and D).

**Fig. 3:**
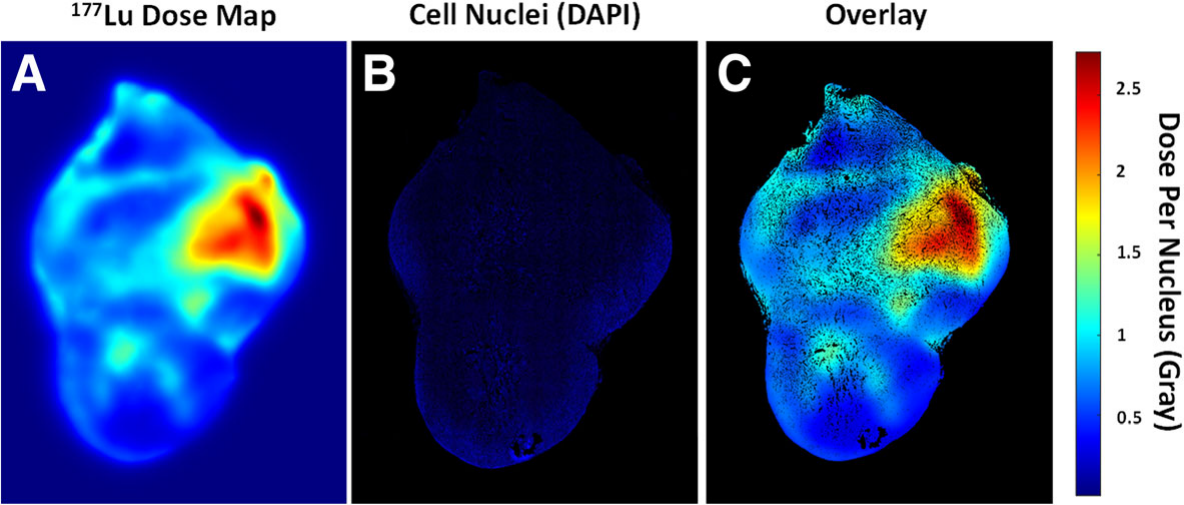
Dose Map: **a**) Map of total dose in Gy from ^177^Lu in all sections onto cells in section 171; **b**) Image of cell nuclei stained with DAPI; **c**) Overlay of dose map onto the cell nucleus image. The black areas in **b** and **c** are areas of low cell density that therefore have no DAPI stain

The overall dose map generated from the multitude of dose contributions onto section 171 (Fig. 3a) was overlaid onto the cell nucleus distribution image (3B) and the dose to each cell nucleus was calculated (3C). The maximum dose per nucleus over the 24 h period was ~2.5 Gy, and the average dose for all nuclei in the section was 0.84 Gy, which is just slightly lower than the average dose calculated by MIRD (0.91 Gy) or by dose kernels for a single point source (1 Gy). This slight discrepancy is due at least in part because some of the dose is distributed outside the tumor region, especially from nanoparticles along the tumor edge. Heterogeneous nanoparticle distribution could also lead to a mean absorbed dose in a section of the tumor that differs from the absorbed dose throughout the entire tumor. Nonetheless, this value, calculated from the dose contributions of many small and heterogeneously distributed point sources, is similar to the average dose calculated from the standard methods described above, suggesting that our novel method is indeed accurate.

**Fig. 4:**
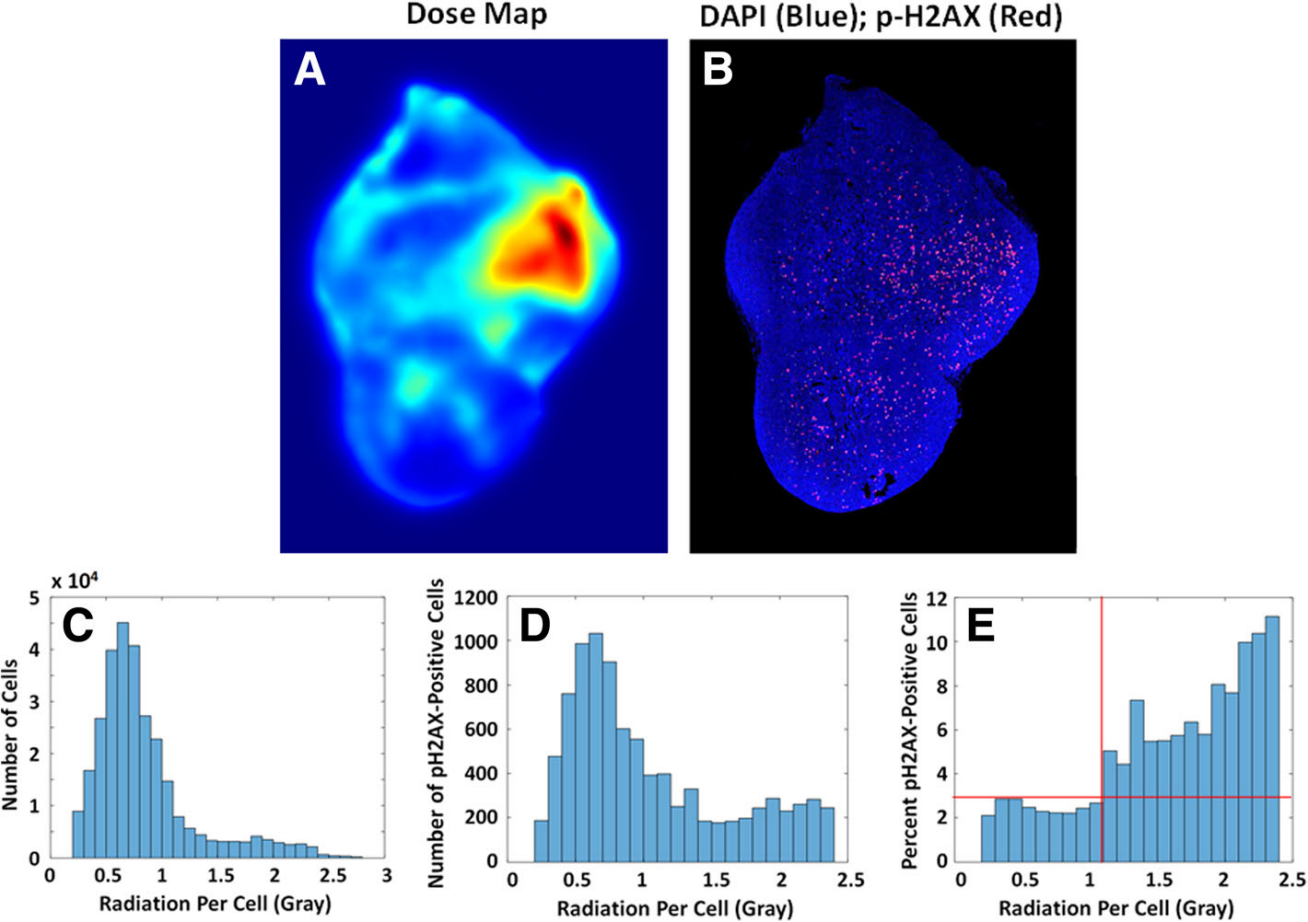
Correlating Dose Map with Cytotoxicity: **a**) ^177^Lu Dose Map; **b**) Distribution of p-H2AX positive cells; *Blue* = DAPI; *Red* = p-H2AX immunostain; **c**) Histogram showing the number of cells that received a given dose of radiation according to the dose map; **d**) Number of p-H2AX positive cells that received a given dose; **e**) Percent of p-H2AX positive cells that received a given dose, taken from bins with > 1000 total cells as seen in **c**. As cells received a higher dose of radiation, a higher percent of those cells stained positive for DNA double-stranded break repair, beginning around 1 Gy over 24 h

To further validate this method of calculating heterogeneous microdosimetry, we removed the coverslip from the middle section and re-stained the tissue for p-H2AX using immunofluorescence. The re-stained section was imaged for DAPI and p-H2AX (Fig. 4b) and overlaid on the dose map (4A) in order to correlate p-H2AX distribution with radiation dose. Figure 4c shows a histogram of the total number of cells that received a certain dose of radiation (bin size = 0.1 Gy), and Fig. 4d shows the total number of p-H2AX positive cells that received a certain dose. Figure 4e combines these two histograms to show that cells which received a higher dose according to our model also had a higher percentage of p-H2AX positive cells. This important figure shows a positive correlation between our model of dose distribution generated from the actual heterogeneous deposition of radiation sources, and the experimentally determined effect of the incident radiation on the cells (H2AX phosphorylation). The figure indicates that radiation-induced H2AX phosphorylation occurs in some cells at a dose as low as approximately one Gy, with an increased dose inducing p-H2AX in an increased percentage of cells.

**Fig. 5:**
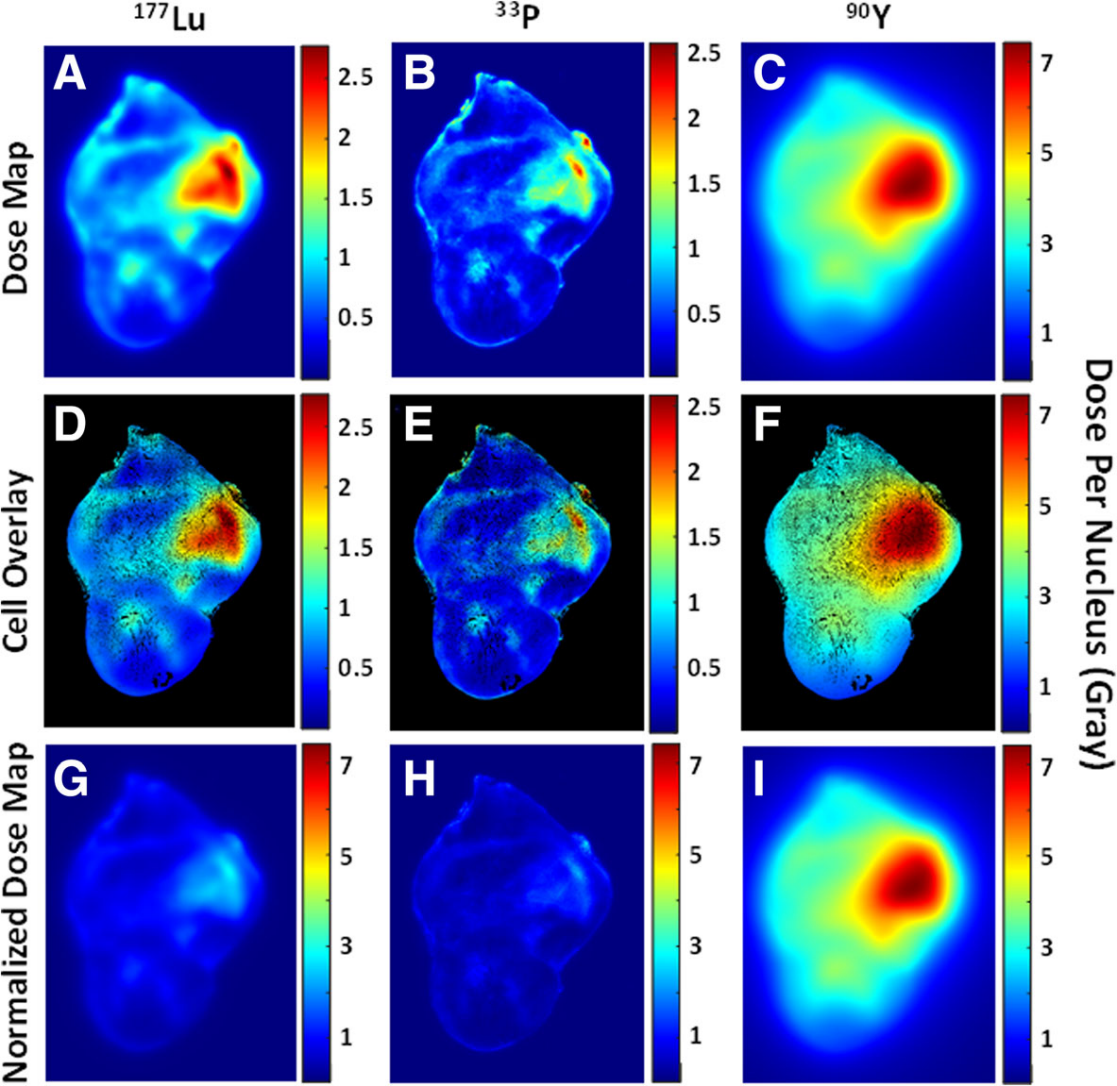
Dose Map for ^177^Lu, ^33^P, and ^90^Y given identical source distribution: **a**-**c**) Dose maps and individual scale bars (in Gy) for ^177^Lu, ^33^P, and ^90^Y, respectively; **d**-**f**) Cell distribution overlay for dose maps in **a**-**c**; **g**-**i**) Dose maps for ^177^Lu, ^33^P, and ^90^Y using the same scale bar min and max

While the nanoparticles used in this study were loaded with ^177^Lu, we can imagine that other nuclides could potentially be loaded into the nanoparticles instead*. In this example, the cellular response (e.g. p-H2AX) to the nanoparticle and dose distribution is only valid for* ^*177*^ *Lu, but the measured nanoparticle distribution can be used to compare how the dose kernels of different nuclides may affect the dose distribution and dose rate to the cells in section 171.* The beta emissions from the clinically used ^90^Y have more energy and thus a longer path length in tissue; in this case, areas with a higher nanoparticle density may be able to deposit dose onto faraway cells that are located in areas of low nanoparticle density. Of course, this may also lead to a higher dose onto healthy tissue adjacent to the tumor, especially from ^90^Y deposited near the tumor edge. In contrast, the beta emissions from the radionuclide ^33^P deposit nearly all of their energy very close to the source, leading to very high radiation doses only in areas of high nanoparticle density. Figure 5a, b, and c show the dose maps and individual scale bars (in Gy) for ^177^Lu, ^33^P, and ^90^Y, respectively, and Fig. 5d, e, and f show the overlay of cell distribution for these dose maps. By comparing the scale bars, one can see that ^90^Y not only provides a more homogeneous dose throughout the left side of tumor section, but also imparts a much larger dose given the same amount of radioactivity (in Bq). This is shown more clearly in Fig. 5g, h, and i, which show the dose maps for ^177^Lu, ^33^P, and ^90^Y using the same scale bar min and max.

**Fig. 6:**
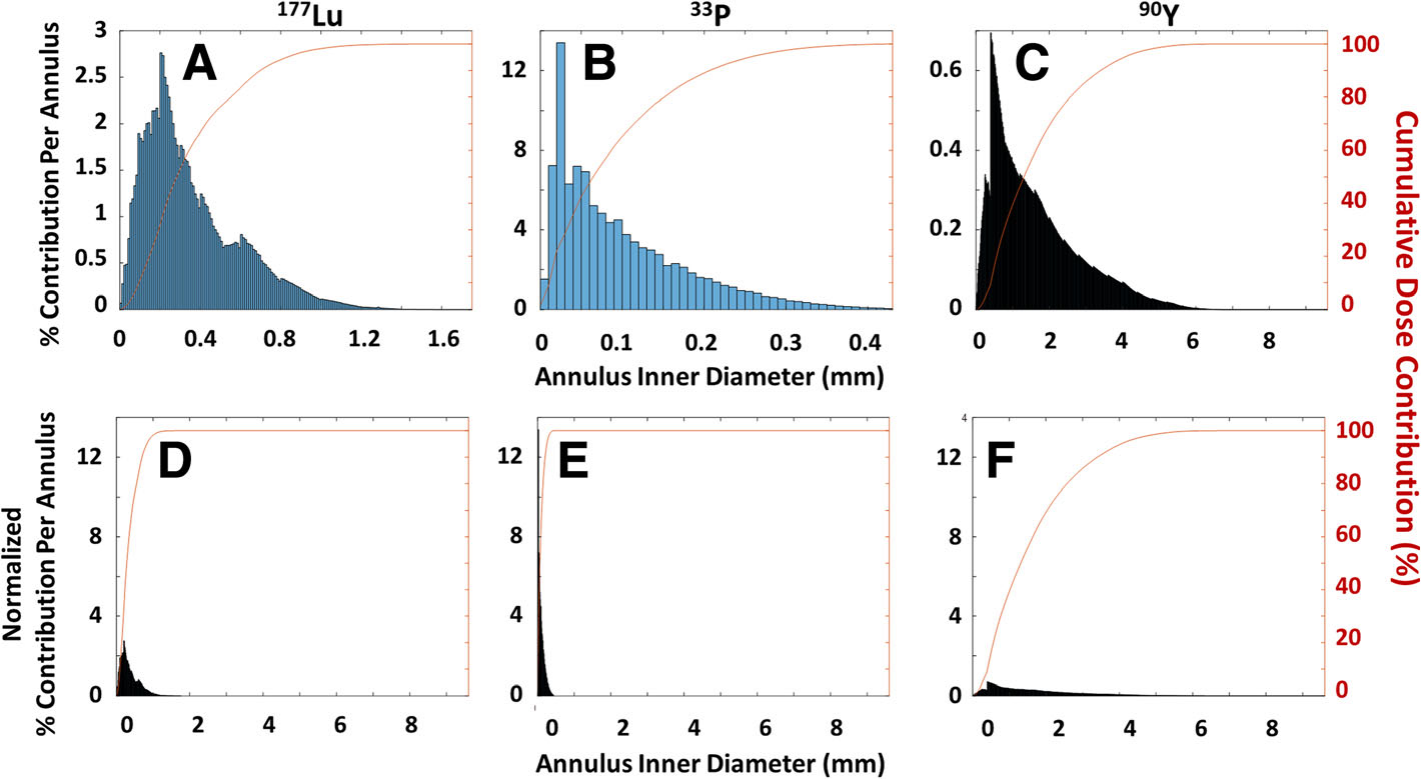
Dose Contributions for ^177^Lu, ^33^P, and ^90^Y at Different Distances from a Given Cell: **a**-**c**) Contributions to a cell’s total absorbed dose from ^177^Lu, ^33^P, and ^90^Y, respectively; **d**-**f**) Contributions with normalized x- and y-axes

We were also interested in changing our perspective to the viewpoint of the cell. We asked what fraction of the total dose to a cell was contributed by radiation sources at different distances away from that cell. For example, at distances very close to a cell, there may be very few particles because the annular volume is small, but the dose contribution from each particle is very high. At distances very far away from the cell, the annular volume is much larger and contains many more particles which each contribute a much smaller dose to that cell. We separately compared the dose contributions from ^177^Lu, ^33^P, and ^90^Y at different distances from that cell in Fig. 6a, b, and c, respectively. The vertical bars represent the percent of the total dose contributed by each annulus, while the red curve across the graph represents the cumulative dose from all annuli as r increases. In Fig. 6d, e, and f, the x- and y-axes were normalized to directly compare the three nuclides. The resulting histograms show that although the individual particles closest to the cells each provide a large dose, the cumulative dose from many particles in a slightly larger (and further away) annulus contribute the most to the cell’s dose. This is true for all three nuclides tested. We also see that nearly 100% of the dose to a cell from ^177^Lu, ^33^P, and ^90^Y is provided by particles within radii of 1 mm, 0.3 mm, and 5 mm, respectively.

## Discussion

Herein, we have shown a novel method for calculating tumor microdosimetry by measuring the actual heterogeneous distribution of nanoparticles carrying the radionuclide ^177^Lu. We have done this by manually slicing hundreds of adjacent tumor sections and generating a 3D stack of the nanoparticle distribution within the tumor. This model has been validated in several different ways: the radioactivity (in Bq) summed over each imaged section agrees with total activity in the whole tumor immediately after resection, the average absorbed dose in Gy according to the model is similar to the average absorbed dose according to both MIRD and single point source dose kernel calculations, and the percentage of p-H2AX-positive cells increases with increasing dose. The dose found to initiate radiation-induced H2AX phosphorylation was approximately one Gy, which is similar to doses in published articles which correlate H2AX phosphorylation to ^177^Lu [31] and x-ray [32] treatment across several cell types *in vitro*. Because externally delivered x-ray doses are given over a short time to induce acute damage, in contrast to β-emitters such as ^177^Lu which provide their dose continuously, x-ray-mediated H2AX phosphorylation is induced at lower doses and normalizes more rapidly [33, 34].

The largest preclinical advantage of this method may be at the interface of tumor dosimetry and tumor biology. The resolution of fluorescent microscopy is orders of magnitude higher than techniques such as SPECT and PET, and fluorescence imaging has long been used to learn about tumor biology as well as drug delivery. The high resolution of these images allowed us to overlay our dose map onto individual p-H2AX-positive cells to quantify dose response, an approach that can be extended to quantify the dose to specific areas such as blood vessels, tumor nests, or hypoxic regions. These methods could be used to study how different cell types and tumor conditions, such as hypoxia, respond to radiation *in vivo.* Similarly, one could examine how differences in the penetration of therapeutic nanoformulations though vasculature or stroma affects the dose rate to tumor cells, or how targeting ligands on nanoparticles affect tumor dosimetry. In these cases, investigation is only limited by image resolution and the accuracy of dose kernels at short distances.

While our data suggests that this approach was successful, there may be ways to recreate this experiment using a less tedious and manually intensive approach. Light sheet microscopy is a so-called volume imaging technique that can image intact (and cleared [35]) organs as large as 1,000 mm^3^ by focusing the incident light in a thin sheet perpendicular to the objective lens, and then moving the light sheet in the z direction to excite fluorophores in each adjacent plane. The entire tissue can be imaged and stitched together in this manner, generating a 3D reconstruction of the tissue without having to cut the tissue into sections. Not only does this method reduce the manual labor associated with cutting and mounting hundreds of adjacent tissue sections, but it also avoids the potential artifacts generated from the stretching or folding of tissue sections as they are cut. The biggest downside to light sheet microscopy—and volume imaging in general—is actually the issue of data storage. One 1,000 mm^3^ 3D image captured with high-resolution light sheet technology can require over 30 terabytes of data per color channel [35], which not only necessitates a huge amount of storage capacity, but also robust computing power when running the designated algorithm. In contrast, the 342 downsampled (10 μm^3^ voxel) images that were used in this current research were less than 100 Megabytes combined.

## Conclusions

The work presented here represents a departure from standard methods of calculating dosimetry, but the data show that this method does indeed accurately model the heterogeneous intratumoral distribution of nanoparticles with very high resolution while also showing a positive correlation with experimentally determined double-stranded break repair. While it is easy to qualitatively observe the distribution of fluorescent nanoparticles in a tumor, the ability of this model to quantify the absorbed dose and dose response in different intratumoral regions allows one to investigate how source deposition in different tumor areas can affect dose and cytotoxicity, as well as how characteristics of the tumor microenvironment, such as hypoxia or high stromal areas, may affect the potency of a given dose.

## Additional files

Additional file 1: MIRD calculations and Pharmacokinetic calculations. Contains Table S1 and Figure S1 (177Lu-LCP Pharmacokinetics), Figure S2 (Overall dose rate for 177Lu-LCP), and Figure S3 (Fraction of Volume Populated by Cell Nuclei). (DOCX 2072 kb)
Additional file 2: Standalone File for Table S1: 177Lu-LCP Pharmacokinetics. The fraction of 177Lu-LCP that has left circulation at each time point is tabulated.(PNG 12 kb)
Additional file 3: Standalone File for Figure S1: 177Lu-LCP Pharmacokinetics. Graphical representation of 177Lu PK shown in Table S1. The fast distribution phase is modeled by a linear equation (Eq 5) and the slower elimination phase is modeled by a logarithmic equation (Eq 6). (PNG 99 kb)
Additional file 4: Table S2 (Dose Kernels for 177Lu, 90Y, and 33P) and S3 (Interpolated Dose Kernels for 177Lu, 90Y, and 33P). (XLSX 78 kb)
Additional file 5: Table S4: Depiction of Dose Kernel Interpolation. (PNG 58 kb)
Additional file 6: Standalone File for Figure S3: Fraction of Volume Populated by Cell Nuclei. A) 10x magnification image of DAPI-stained nuclei in an area in section 171; B) Binary representation of nuclear distribution used to quantify nuclear density. Cell nuclei populated ~40% of the total image area. Nuclear radius measured to be an average of ~5 μm. (PNG 401 kb)

## Abbreviations

^177^Lu: ^177^Lutetium
^177^LuCl3: ^177^Lutetium Chloride
^177^Lu-LCP: ^177^Lu-loaded Lipid-Calcium-Phosphate Nanoparticle
^33^P: ^33^Phosphorus
3D: 3-Dimensional
Y: ^90^Yttrium
Bq: Becquerel
DOPA: 1,2-dioleoyl-*sn*-glycero-3-phosphate
DOTAP: 1,2-dioleoyl-3-trimethylammonium-propane
DSPE-PEG2000: N-(Carbonyl-methoxypolyethyleneglycol 2000)-1,2-distearoyl-sn-glycero-3-phosphoethanolamine, sodium salt
Gy: Gray
MIRD: Medical Internal Radiation Dose
PET: Positron Computed Tomography
RFU: Relative Fluorescence Units
SPECT: Single Photon Emission Computed Tomography
UNC: The University of North Carolina at Chapel Hill
