# Supplementary material for: A dosimetric model for the heterogeneous delivery of radioactive nanoparticles In vivo: a feasibility study
Source: Radiat Oncol. 2017 Mar 17;12:54. doi: 10.1186/s13014-017-0794-z (PMC5356254; doi:10.1186/s13014-017-0794-z)
Supplement: Additional file 1: — MIRD calculations and Pharmacokinetic calculations. Contains Table S1 and Figure S1 (177Lu-LCP Pharmacokinetics), Figure S2 (Overall dose rate for 177Lu-LCP), and Figure S3 (Fraction of Volume Populated by Cell Nuclei). (DOCX 2072 kb) [file 13014_2017_794_MOESM1_ESM.docx]

**MIRD Calculations:**

The MIRD Committee has defined the mean absorbed dose (D̅) to an organ as the integral of the dose rate (Ḋ) over time (t) (Eq.1), where the dose rate equals the accumulated activity (A) multiplied by the absorbed dose in the target tissue (T) per unit of activity in the source tissue (S), denoted as DF (T🡨S) [[1-5](#_ENREF_1)] (Eq.2). The following calculations describe dosimetry for a subcutaneous tumor after intravenous injection of ^177^Lu-LCP, which has a mean energy from beta particles, conversion electrons, and Auger electrons of 147 keV [[5](#_ENREF_5)]. For this analysis, the absorbed dose from gamma photons released by ^177^Lu was not considered.

Strictly speaking, ^177^Lu present at the edge of the tumor will impart a portion of its energy into the surrounding tissue, and off-target ^177^Lu present in the surrounding tissue and very close to the tumor will impart a portion of its energy into the tumor. In this approximation, it is assumed that all energy released by the delivered ^177^Lu is absorbed by the tumor, and no energy from off-target ^177^Lu is absorbed by the tumor. These calculations quantify the average absorbed dose to the entire tumor according to the equations below:

1. $\bar{D}=\int_{0}^{86,400s} \dot{D}\left( t \right)\mathrm{dt}$
2. $\dot{D}(t)= A\left( t \right)*DF (TS)$

where:

$\bar{D}=Gy= \frac{Joules (J)}{kg}$

$\dot{D}=\frac{J}{second (s)} or \frac{Gy}{s}$

$A=Becquerel (Bq)=\frac{decays}{s}$

$\mathrm{DF}\left( TS \right)=\frac{Gy}{decay}$

The tumor studied for these calculations was a subcutaneously inoculated UMUC3/3T3 tumor that was measured to weigh **0.13 grams** and contain **1.63 μCi** of activity from ^177^Lu when it was dissected from a mouse at t = 24 h after injection of ^177^Lu-LCP. Taking radioactive decay into account (^177^Lu t_1/2_ = 6.71 days = 579,744s), this activity corresponds to an activity of **1.81** **μCi** at t = 0 h according to Eq 3. Later, this value will be further adjusted to reflect tumor accumulation of ^177^Lu-LCP over time.

(3) $A_{t}=A_{0}e^{-\gamma t}$, where $\gamma=\frac{ln(2)}{t_{\frac{1}{2}}}$

To convert μCi to Gy/s absorbed by the tumor at t = 0, before taking into account the kinetics of tumor accumulation, the following unit conversions were applied:

$$1.81 \mu\mathrm{Ci}* \frac{37,000\frac{decays}{sec}}{\mu\mathrm{Ci}}*\frac{147 KeV}{decay}*\frac{1.6E\left( -16 \right) J}{KeV}*\frac{1}{0.0001315 kg tumor}=\boldsymbol{0.000012}\frac{\boldsymbol{Gy}}{\boldsymbol{s}}$$

This conversions yields the following values at t = 0 for $\dot{D}$, A, and $\mathrm{DF}\left( TS \right)$:

$\dot{\mathbf{D}}\boldsymbol{=}\boldsymbol{0.000012}\frac{\boldsymbol{Gy}}{\boldsymbol{s}}\boldsymbol{=1.6}\boldsymbol{E}\left( \boldsymbol{-9} \right)\frac{\boldsymbol{J}}{\boldsymbol{s}}$ (units of J/s used in Eq 4 and converted to Gy/s)

$\boldsymbol{A=66970}\frac{\boldsymbol{decays}}{\boldsymbol{s}}$

$\mathbf{DF}\left( \mathbf{T}\mathbf{S} \right)\mathbf{=1.8E (-10)}\frac{\boldsymbol{Gy}}{\boldsymbol{decay}}$

The dose rate in the tumor may change over time for many reasons. Two of these reasons were accounted for in these calculations:

1. Tumor dose rate will decrease over time due to radioactive decay. Activity at time t can be calculated according to Eq 3. The dose to this tumor with respect to decay at time t is therefore:

(4) $\dot{D}(t) =1.6E-9*e^{-1.2E-6*t}$

1. The tumor accumulation and pharmacokinetics of systemically injected ^177^Lu-LCP affect dose rate because at early times after injection, much of the activity is still in circulation. Just after injection, the tumor begins accumulating nanoparticles and therefore activity. By measuring ^177^Lu-LCP circulation pharmacokinetics in n = 5 mice, the percentage of injected nanoparticles still in circulation as a function of time was determined. In order to estimate the amount of activity within the tumor at 0 < t < 24 h, it was assumed that the tumor accumulation was proportional to the fraction of the injected dose that had left circulation at that time, with the maximum tumor accumulation occurring at t = 24h (Table S1 and Figure S1). For example, at t = 4 h, the pharmacokinetic experiment showed that an average of 87% of ^177^Lu-LCP had been cleared from circulation, so it was approximated that the tumor in question contained 87% of the activity present at 24 h (the time at which the tumor was dissected). Because ^177^Lu is a trivalent cation, it tends to remain in the initial organ of deposition, suggesting that this method to approximate the time dependent accumulation of activity is appropriate.

Table S1: ^177^Lu-LCP Pharmacokinetics**. The fraction of ^177^Lu-LCP that has left circulation at each time point is tabulated.**

Figure S1: ^177^Lu-LCP Pharmacokinetics**. Graphical representation of ^177^Lu PK shown in Table S1. The fast distribution phase is modeled by a linear equation (Eq 5) and the slower elimination phase is modeled by a logarithmic equation (Eq 6).**

Two equations were then calculated to model the two distinct phases of ^177^Lu-LCP PK:

(5) For 0 < t < 1,800s, $y=0.0045t$

(6) For 1,801 < t < 86,400s, $y=0.05\ln\left( t \right)+0.41$

By applying ^177^Lu-LCP PK to the radioactive decay equation, one overall dose rate curve was generated (Figure S2). Two separate equations were used to describe the dose rate:

(7) For 0 < t < 1,800s, $\dot{D}\left( t \right)=7E-13*t$

(8) For 1,801 < t < 86,400s, a high order polynomial was used to best model the curve: $\dot{D}\left( t \right)=3.2E-34*t^{5}\boldsymbol{-}9.4E-29*t^{4}+1.1E-23*t^{3}-6.4E-19*t^{2}+1.9E-14*t+1.1E-9$

Solving Eq.1 using this piecewise function therefore yields the following value for total absorbed dose over the first 24h after ^177^Lu-LCP intravenous injection:

$$\bar{D}=\int_{0}^{86,400s} \dot{D}\left( t \right)\mathrm{dt}=\boldsymbol{0.91 Gy}$$


Figure S2: Overall dose rate for ^177^Lu-LCP**. Graphical representation of overall dose rate. To achieve the best fit, two trend lines were generated. The initial linear equation modeled dose from 0 < t < 1,800s, and a high order polynomial modeled 1,800 < t < 86,400s with an R^2^ value of 0.999.**

**Figure S3: Fraction of Volume Populated by Cell Nuclei: A) 10x magnification image of DAPI-stained nuclei in an area in section 171; B) Binary representation of nuclear distribution used to quantify nuclear density. Cell nuclei populated ~40% of the total image area. Nuclear radius measured to be an average of ~5 μm.**

1. Mjekiqi, E., *Estimation of the absorbed dose to patients treated with 177LuDotatate with regards to the long-term retention and radionuclide impurity in the form of 177mLu* in *Medical Radiation Physics*2012, Lund University: lup.lub.lu.se/student-papers/record/3364795/file/3364801.pdf. p. 111.

2. Cremonesi, M., et al., *Dosimetry in Peptide radionuclide receptor therapy: a review.* J Nucl Med, 2006. 47(9): p. 1467-75.

3. Howell, R.W., et al., *The MIRD perspective 1999. Medical Internal Radiation Dose Committee.* J Nucl Med, 1999. 40(1): p. 3S-10S.

4. Gulec, S.A., G. Mesoloras, and M. Stabin, *Dosimetric techniques in 90Y-microsphere therapy of liver cancer: The MIRD equations for dose calculations.* J Nucl Med, 2006. 47(7): p. 1209-11.

5. Schmitt, A., et al., *Biodistribution and dosimetry of 177Lu-labeled [DOTA0,Tyr3]octreotate in male nude mice with human small cell lung cancer.* Cancer Biother Radiopharm, 2003. 18(4): p. 593-9.
