# Supplementary figures and images for: A dosimetric model for the heterogeneous delivery of radioactive nanoparticles In vivo: a feasibility study
Source: Radiat Oncol. 2017 Mar 17;12:54. doi: 10.1186/s13014-017-0794-z (PMC5356254; doi:10.1186/s13014-017-0794-z)

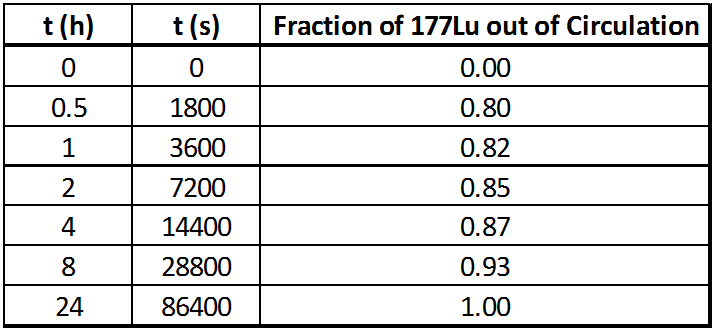

Supplement: Additional file 2: — Standalone File for Table S1: 177Lu-LCP Pharmacokinetics. The fraction of 177Lu-LCP that has left circulation at each time point is tabulated.(PNG 12 kb) [file 13014_2017_794_MOESM2_ESM.png]

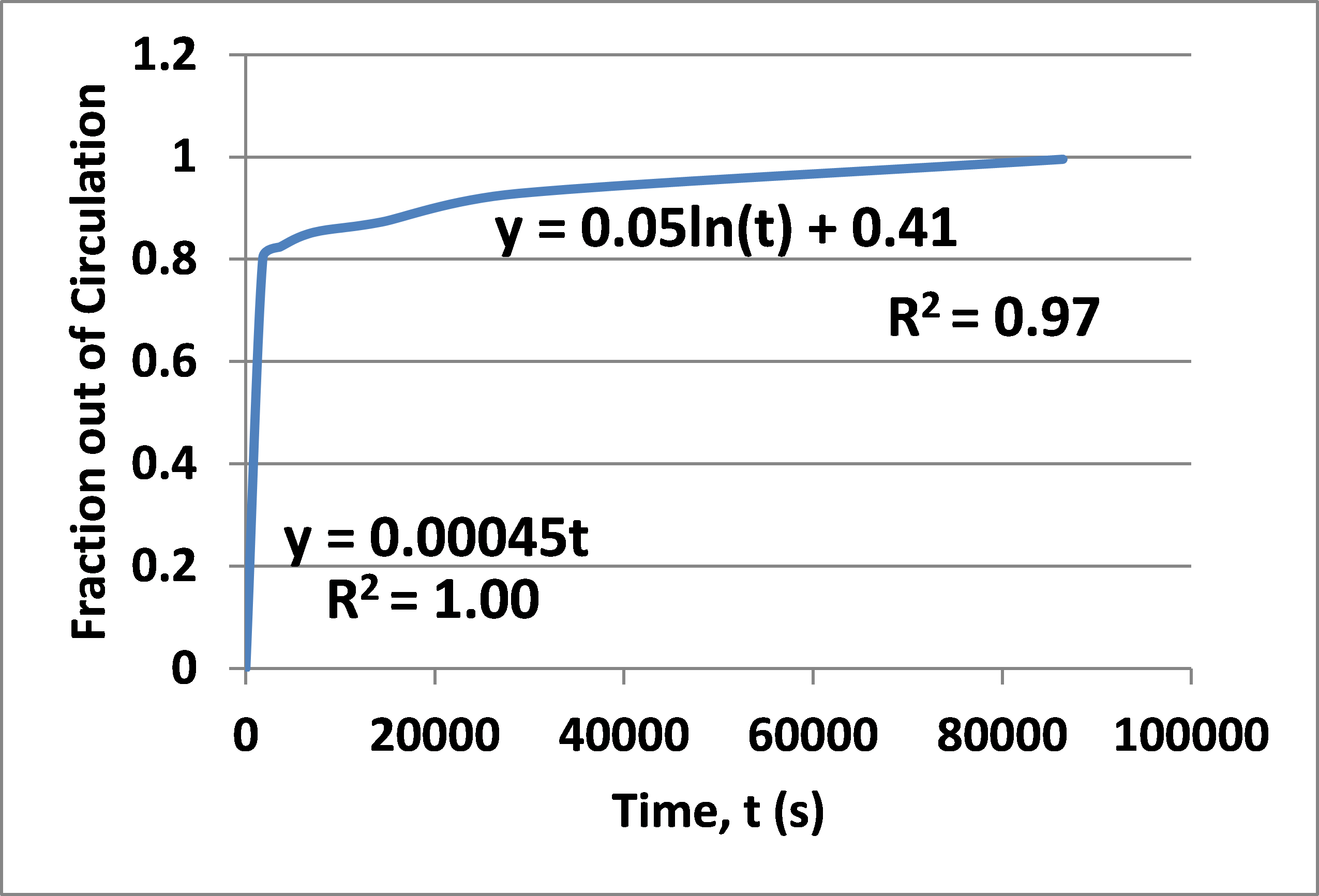

Supplement: Additional file 3: — Standalone File for Figure S1: 177Lu-LCP Pharmacokinetics. Graphical representation of 177Lu PK shown in Table S1. The fast distribution phase is modeled by a linear equation (Eq 5) and the slower elimination phase is modeled by a logarithmic equation (Eq 6). (PNG 99 kb) [file 13014_2017_794_MOESM3_ESM.png]

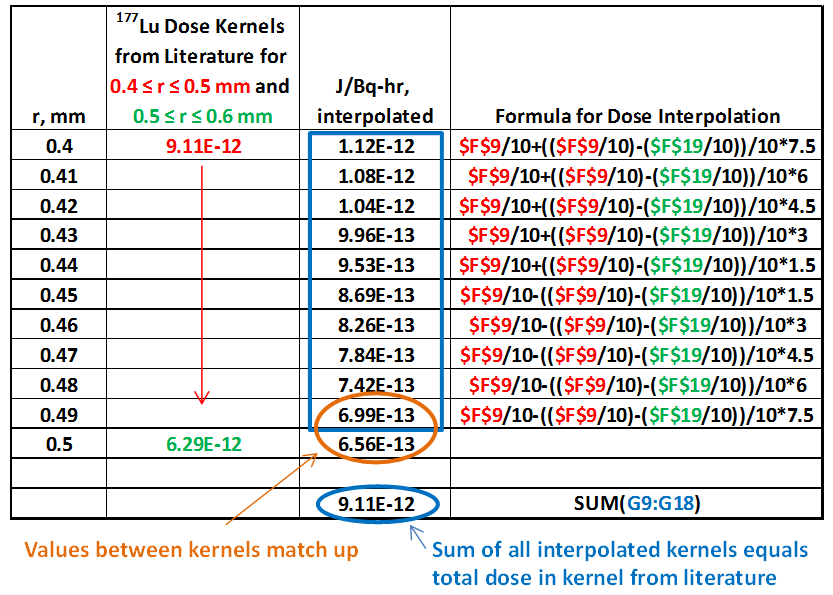

Supplement: Additional file 5: — Table S4: Depiction of Dose Kernel Interpolation. (PNG 58 kb) [file 13014_2017_794_MOESM5_ESM.png]

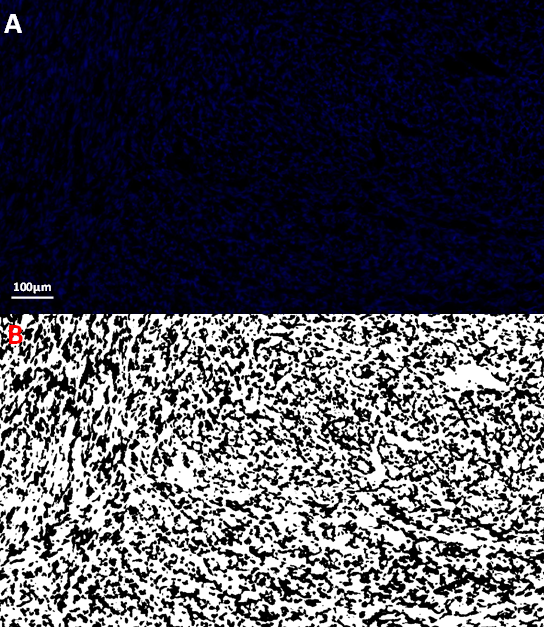

Supplement: Additional file 6: — Standalone File for Figure S3: Fraction of Volume Populated by Cell Nuclei. A) 10x magnification image of DAPI-stained nuclei in an area in section 171; B) Binary representation of nuclear distribution used to quantify nuclear density. Cell nuclei populated ~40% of the total image area. Nuclear radius measured to be an average of ~5 μm. (PNG 401 kb) [file 13014_2017_794_MOESM6_ESM.png]
